# Lipoteichoic acid mediates binding of *Streptococcus pneumoniae* and influenza A virus

**DOI:** 10.1128/msphere.00504-25

**Published:** 2025-11-28

**Authors:** Trevor Penix, Jenna Favazza, Jason W. Rosch, Hannah M. Rowe

**Affiliations:** 1 Department of Host-Microbe Interactions, St Jude Children’s Research Hospital, Memphis, Tennessee, USA; 2 Department of Microbiology, Oregon State University 2694, Corvallis, Oregon, USA; University of Michigan, Ann Arbor, Michigan, USA

**Keywords:** *Streptococcus pneumoniae*, influenza, lipoteichoic acid

## Abstract

Synergy between influenza A virus (IAV) and *Streptococcus pneumoniae* is a long-recognized and clinically important problem. Recent work has demonstrated that IAV particles can directly bind to the bacterial surface and that bacterial-viral complexes exhibit enhanced bacterial colonization and invasive disease, increased viral environmental survival leading to increased efficacy of airborne transmission, and enhanced vaccine response to both pathogens over simultaneous co-infection without direct interactions. However, the molecule(s) responsible for mediating the direct interaction are yet to be characterized. In this study, we demonstrate that the broadly conserved Gram-positive bacterial cell wall glycan lipoteichoic acid (LTA) is one of the molecules that can mediate this interaction. This interaction between viral particles and bacterial cell-envelope glycans is also demonstrated in interactions between enteric viruses and enteric bacteria, suggesting a conserved mechanism of trans-kingdom interactions. We show that LTA will compete for binding between IAV and *S. pneumoniae*, that disruption of genes responsible for LTA presentation at the cell surface will reduce viral binding, and that viral neuraminidase can bind LTA. This work adds to the growing body of literature on direct bacterial-viral interactions between human-associated bacteria and pathogenic viruses and can provide novel insights into the lethal synergy of influenza-pneumococcal co-infections.

**IMPORTANCE:**

Co-infection between influenza A virus (IAV) and *Streptococcus pneumoniae* leads to severe disease. Recently, it was demonstrated that IAV particles can bind to the surface of bacterial cells and that direct interactions will enhance both bacterial and viral pathogenesis as well as immune responses to each pathogen. However, it is unclear what bacterial and viral components are responsible for the interaction. We demonstrate that a carbohydrate component of the bacterial cell wall can bind to IAV particles. This is similar to direct interactions observed between enteric viruses and cell wall components of enteric bacteria. This work adds to the body of knowledge about trans-kingdom interactions between human-associated bacteria and human pathogenic viruses, as well as providing novel insights into the serious clinical problem of influenza-pneumococcal synergy.

## INTRODUCTION

Viral coinfection is a well-established risk factor for subsequent bacterial pneumonia. During the devastating 1918 influenza pandemic, reports show that morbidity and mortality were largely driven by secondary bacterial infection (1). These bacterial infections are still found in fatal cases of influenza in modern pandemics, emphasizing the importance of these bacterial-viral interactions (2). One of the best-characterized examples is the synergy observed between influenza virus and *Streptococcus pneumoniae*, whereby prior viral challenge dramatically enhances susceptibility to secondary bacterial pneumonia and bacteremia. *S. pneumoniae* and human influenza A viruses (IAV) demonstrate interkingdom synergy in terms of exacerbating morbidity and mortality of pneumonia, both clinically and in murine models of infection (3–5). The underlying mechanisms of this synergy are complex and multifactorial, with bacterial, viral, and host immune factors playing important roles in the enhanced susceptibility (6). Most described interactions are indirect, with the viral infection preparing a more favorable environment for opportunistic bacterial secondary infection. For example, influenza is known to impair the immune system by depleting alveolar macrophages (7), effectively disarming a critical component of the host immune defense network in the respiratory tract. Additionally, the cellular damage caused by an influenza infection (6) has been shown to release nutrients (8), in addition to direct action of viral neuraminidase in providing a carbon source to bacteria (9), uncovering binding sites (10) for bacteria, and altering mucociliary clearance of pneumococci from the trachea (11). Specific host-side molecular interactions have also been outlined, as influenza infection can boost bacterial adherence by upregulating host proteins known for bacterial binding. In the case of *S. pneumoniae*, influenza has been shown to upregulate host platelet-activating factor receptor, or PAFR, a known binding partner for pneumococcal phosphorylcholine (12). These mechanisms provide multiple pathways that contribute to the heightened morbidity and mortality observed in secondary bacterial infections.

More recent work has suggested an additional mechanism of bacterial-viral synergy, whereby direct physical interactions between IAV and bacterial pathogens impact several aspects of both bacterial and viral biology. Influenza has been shown to directly adhere to various respiratory bacteria, including the secondary bacterial pathogen *S. pneumoniae* (13, 14). These interactions enhanced bacterial pathogenesis with the pneumococci, with adherent virus exhibiting enhanced binding to tissue culture, enhanced murine nasopharyngeal colonization, and increased lethality in mice compared to bacterial infection alone (13). Evidence also supports that co-colonizing bacteria can promote viral stability in the environment in both droplets and aerosols, which may also promote more effective airborne viral transmission (15, 16). Respiratory syncytial virus (RSV) has been shown to directly bind to the pneumococcal surface via penicillin-binding protein 1a, enhancing bacterial adherence to the epithelial surface and mediating more severe disease in murine models of infection (17, 18). This synergism is most pronounced upon simultaneous administration, indicating that RSV is likely a direct coupling partner in the enhanced binding to respiratory cells (18). These interactions with RSV may also extend to several additional bacterial pathogens as expression of the RSV glycoprotein enhances epithelial binding by *Streptococcus pneumoniae* and *Haemophilus influenzae* (19). RSV also mediates *Pseudomonas aeruginosa* binding to epithelial cells from healthy controls and patients with cystic fibrosis (20), while interactions between *S. aureus* proteases and IAV can activate the IAV hemagglutinin (21). These interkingdom interactions are not limited to pathogens of the respiratory tract, as poliovirus has been shown to bind bacteria via LPS—with the complex benefiting the virus via enhanced viral infectivity and increased viral recombination (22)—and bacterial-viral interactions have also been shown for other enteric viruses (23–26).

While several lines of investigation have found evidence of direct interkingdom interactions, the mechanisms underlying the binding interaction have typically been more complicated to discern. Several possibilities for surface features mediating bacterial binding to IAV exist. Previous work has eliminated some options for a bacterial binding partner, as pneumococci of several serotypes, as well as non-capsulated strains, appear to bind IAV particles at similar levels (13, 16), suggesting that the polysaccharide capsule of pneumococcus is not contributing to the interaction with IAV. Given that influenza can bind a variety of bacterial species common to the human upper respiratory microflora (13), we postulated that a common surface structure, such as peptidoglycan or lipoteichoic acid, is involved. One study found that lipopolysaccharide is capable of binding to influenza virions, resulting in destabilization of the virus and loss of infectivity (27). This suggests that some bacterial glycans enhance viral infectivity, while others are detrimental. Finding the exact binding partners between influenza and respiratory bacteria, as well as the consequences of these interactions, will be essential in addressing the synergistic and antagonistic relationships between respiratory viruses and bacterial inhabitants of the upper respiratory tract. In this study, we present data to bridge this gap in understanding by identifying lipoteichoic acid as a mediator for direct bacterial-viral interactions between *S. pneumoniae* and IAV.

## RESULTS

Bacterial-viral interactions between enteric bacteria and viruses are mediated by bacterial cell envelope polysaccharides. Poliovirus and rotavirus have been shown to directly bind to lipopolysaccharide, lipoteichoic acid, and peptidoglycan (22–25). Norovirus has been shown to bind to bacterial envelope glycans of the histo-blood group antigen (26). Here, we wanted to characterize the potential of pneumococcal cell envelope glycans to mediate the direct interactions between *S. pneumoniae* and influenza A virus. The three primary glycan components of the pneumococcal cell envelope that could serve as potential binding targets are capsule, teichoic acid, and peptidoglycan. Prior work demonstrating pneumococcal serotype-independent binding and equivalent binding to acapsular pneumococci (13) indicated a lack of a role of capsule in direct viral binding, leaving teichoic acid and peptidoglycan as the most likely candidates.

**Fig 1:**
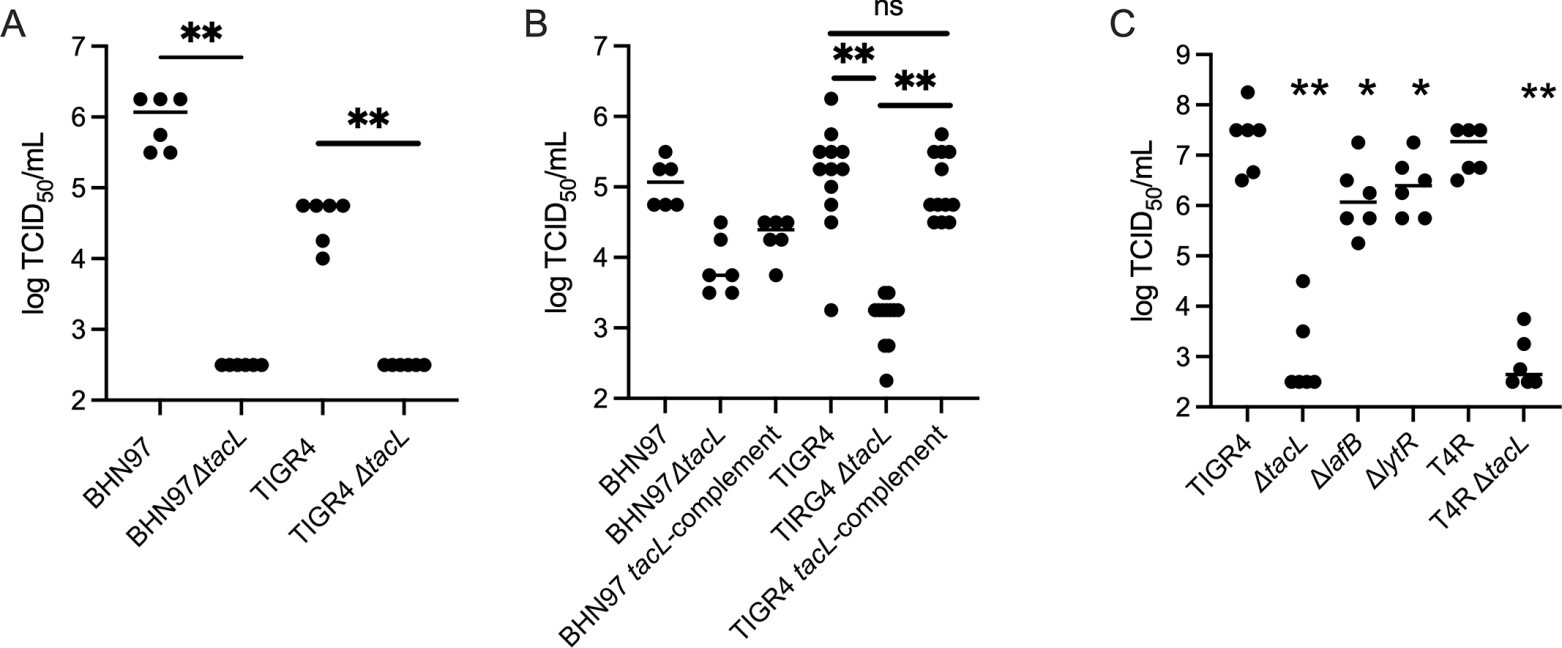
Deletion of pneumococcal LTA expression genes reduces interaction with IAV. Mid-log bacterial cells of indicated strain were incubated with IAV strain PR8 and bound IAV assessed by TCID_50_. (**A**) *tacL* deletion reduces viral binding in strains BHN97 and TIGR4. (**B**) Complementation of *tacL* at an ectopic locus partially complements IAV binding in BHN97 and fully complements IAV binding in TIGR4. (**C**) Deletion of *lafB* and *lytR* also significantly reduces IAV binding to strain TIGR4, and *tacL* dependence of IAV binding is capsule independent as similar results are seen in acapsular T4R strain. *P < 0.05, **P < 0.01, n.s. not significant by Mann-Whitney.

We first sought to answer this question by altering bacterial glycan expression through a genetic approach. Peptidoglycan is necessary for bacterial viability, but unfortunately, could not be assessed by this method. Teichoic acid has two forms: membrane-anchored lipoteichoic acid (LTA) and peptidoglycan-anchored wall teichoic acid (WTA). For *S. pneumoniae*, the carbohydrate portion of LTA and WTA is identical (28). Pneumococci expressing neither WTA nor LTA are non-viable, so it is not possible to generate a completely teichoic acid-null strain to determine viral binding affinity. However, it is possible to delete *tacL* (*SP_1893*), the ligase responsible for attaching the carbohydrate moieties of teichoic acid to the glycolipid anchor to generate LTA (29). This mutant will theoretically only express WTA. As a measure of bacterial-viral binding, we used a simple co-sedimentation assay. Bacterial mutants were co-incubated with influenza virus, followed by a sedimentation and wash step, removing any unbound virus. Any virus remaining in the bacterial pellet was measured by 50% tissue culture infectious dose (TCID_50_). For both BHN97 and TIGR4 strains of pneumococcus, deletion of *tacL* resulted in significantly reduced levels of infectious virus bound to the bacterial pellet (Fig. 1A), suggesting that LTA plays a role in mediating the bacterial-viral interaction. To confirm the role of *tacL*, we restored *tacL* to an ectopic locus using the chromosomal expression platform (30) to complement the deletion in both BHN97 and TIGR4. The *tacL* complement in TIGR4 fully restored viral binding, while the *tacL* complement in BHN97 partially restored viral binding (Fig. 1B).

To further characterize the role of lipoteichoic acid, we also examined the binding of IAV to a *lafB (SP_1075*) deletion. LafB is responsible for the synthesis of the lipid component of LTA, galactosyl-glucosyl-diacylglycerol (31); therefore, a *lafB* deletion will also be deficient in LTA. The binding of IAV to Δ*lafB* was significantly reduced compared to wild-type TIGR4 (Fig. 1C), further supporting the role of LTA in viral binding to *S. pneumoniae*. To examine the role of WTA, we deleted *lytR (SP_1942*), which is responsible for the attachment of teichoic acid precursors to PG (32). We again saw significantly reduced binding of IAV to the Δ*lytR* mutant, compared to the TIGR4 parental strain (Fig. 1C). The Δ*lytR* mutant likely still has some WTA expression due to the compensatory roles played by other LytR-CpsA-Psr family proteins (33, 34). Finally, to confirm that the capsule was not playing a role in viral binding to the bacterial surface, we deleted *tacL* in the T4R acapsular strain and demonstrated an identical loss of viral binding to the Δ*tacL* T4R (Fig. 1C).

**Fig 2:**
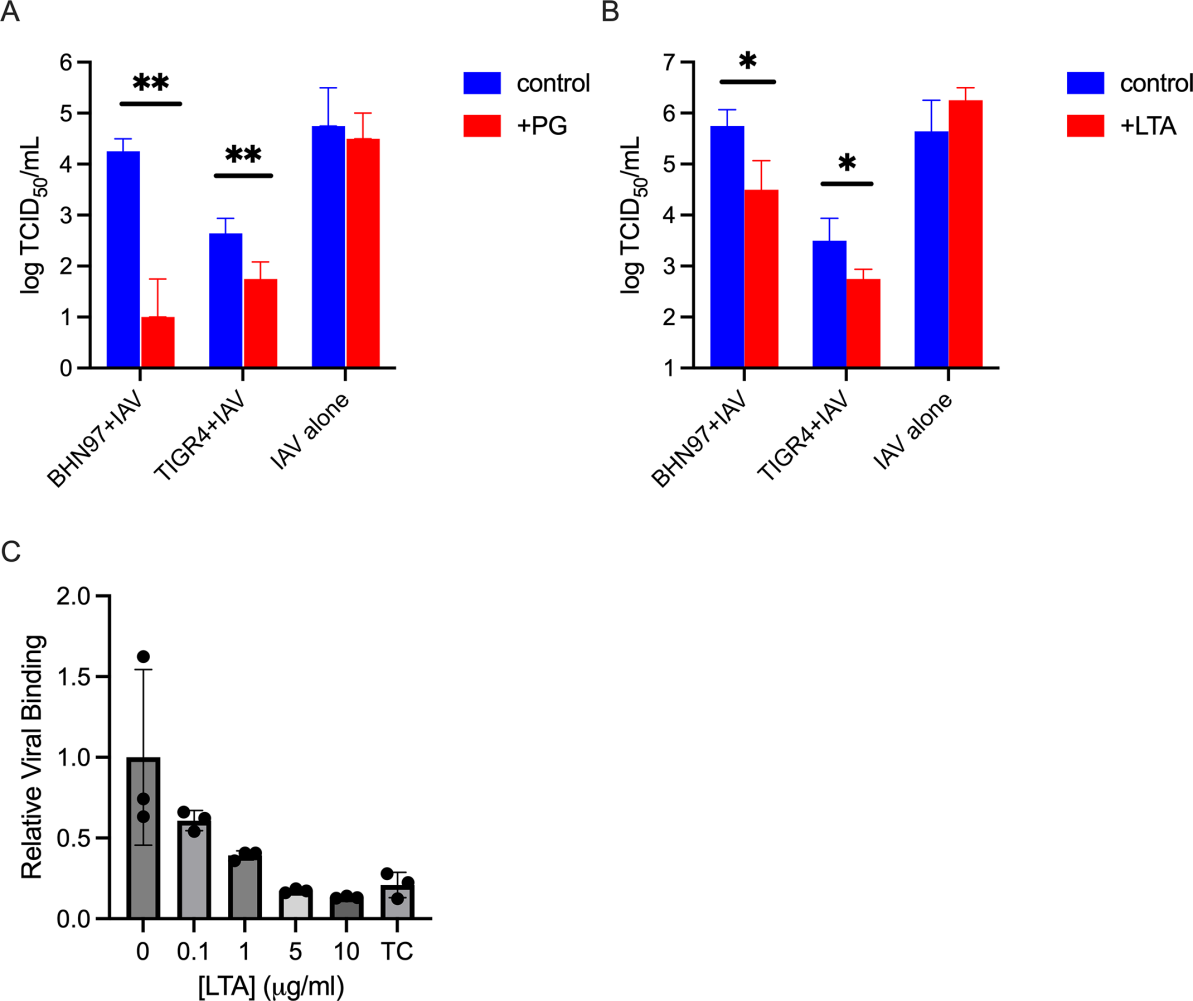
Cell wall glycans compete for IAV binding to *S. pneumoniae*. (**A**) IAV strain PR8 was pre-incubated with 1 µg/mL *B. subtilis* peptidoglycan (PG) or control, followed by incubation with *S. pneumoniae* and bound IAV assessed by TCID_50_ IAV alone samples were assessed by TCID_50_ without incubation with bacteria. (**B**) IAV strain PR8 was pre-incubated with 1 µg/mL *S*. *pyogenes* lipoteichoic acid (LTA) or control, followed by incubation with *S. pneumoniae* and bound IAV assessed by TCID_50_. *P < 0.05, **P < 0.01, n.s. not significant by Mann-Whitney. (**C**) IAV strain A/California.04/2009 was pre-incubated with *S. pneumoniae* strain TIGR4 in the presence of indicated concentration of *S. pyogenes* LTA and bound IAV assessed by qRT-PCR for viral genomic RNA. TC = tube control, representing the amount of viral RNA bound to the plastic tube in the absence of bacterial cells. Correlation plot in Fig. S1.

As an orthogonal method to assess the nature of viral binding, we used a competitive binding assay with purified glycans. The principle of this method is to pre-incubate influenza with excess glycan prior to introducing the virus to bacteria. If the glycan is responsible for bacterial-viral binding, the excess glycan should disrupt the complex formation. Due to the high degree of conservation between Gram-positive peptidoglycan, we used commercially available *Bacillus subtilis* peptidoglycan (PG). For lipoteichoic acid, we utilized commercially available *Streptococcus pyogenes* lipoteichoic acid (LTA). We pre-incubated virus and glycan to allow association, then added to washed pneumococci resuspended in PBS and incubated to form bacterial viral complexes. After centrifugation and washes to remove non-adherent virus, the amount of virus present was determined by TCID_50_. Pre-incubation of IAV with both PG (Fig. 2A) and LTA (Fig. 2B) resulted in significantly less virus bound, demonstrating that an excess of PG or LTA can compete for bacterial binding. In addition, we utilized two strains of *S. pneumoniae*, a serotype 4 (TIGR4) and a serotype 19F (BHN97), to demonstrate that the competitive binding was not serotype or strain-dependent.

We next examined the dose dependency of LTA in competing for bacterial binding to viral particles by performing a co-sedimentation assay with LTA concentrations ranging from 0 to 10 μg/mL and measuring viral binding by qRT-PCR to determine relative viral genome copies associated with bacteria. Both the TCID_50_ and fluorescence-based assay measure infectivity of viral particles bound to bacterial cells, not the binding of viral particles directly. We demonstrate a significant negative correlation (Spearman r = −0.9711) of viral genome copies present in the washed bacterial pellet as the LTA concentration increases (Fig. 2C).

**Fig 3:**
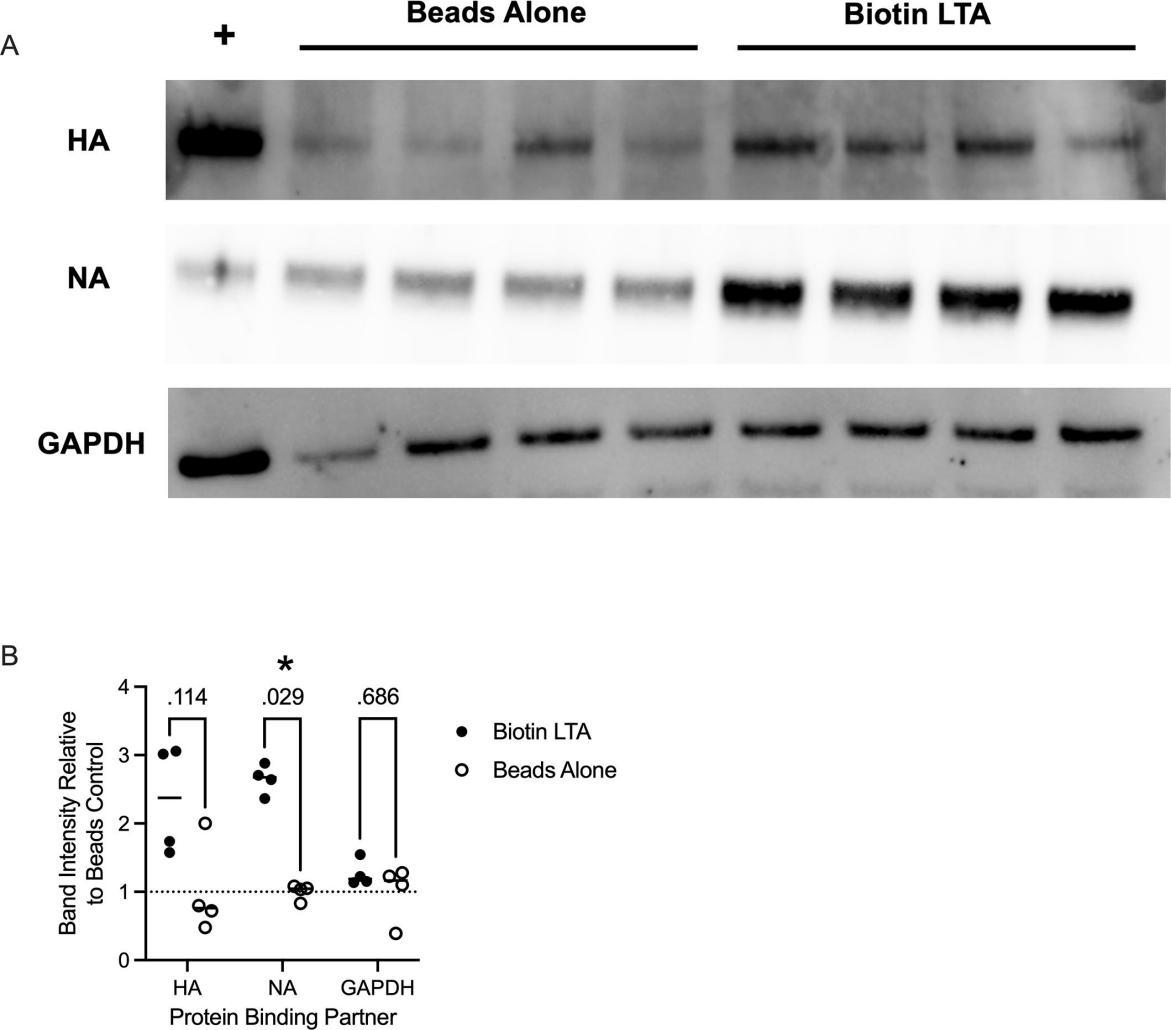
LTA interacts with Viral HA protein. Biotinylated *S. pyogenes* LTA was incubated with a non-specific protein GAPDH, hemagglutinin (HA) from A/California/04/2009 (H1), or neuraminidase (NA) from A/California/04/2009 (N1), followed by pulldown with streptavidin beads, and detected by Western blot (**A**). (Biotin LTA) = pulldown of purified protein after interaction with biotinylated LTA. (Beads alone) = pulldown of purified protein with beads alone. Densitometry measurements (**B**) were taken in ImageJ and normalized based on the average of “Beads alone” measurements (35). *P < 0.05 by Mann-Whitney. Uncropped blots are available as Fig. S2.

To determine the viral partner responsible for the interaction, we examined the interactions between LTA and purified viral surface proteins hemagglutinin (HA) and neuraminidase (NA). Given that HA is the receptor for host binding, we suspected that it is likewise responsible for binding lipoteichoic acid. While the native target of HA, sialic acid, is absent in LTA, other sugar structures may partially bind. NA also interacts with sialic acid, so we assessed its ability to interact with LTA as well. We demonstrated that biotinylated LTA from *S. pyogenes* significantly interacts with NA through a bead-based pulldown, as shown in Fig. 3. While not significant, there appears to be a trend for LTA binding HA as well. Meanwhile, unrelated control protein GAPDH does not interact with LTA. These findings support that sialic acid receptors of HA and NA may be partially interacting with other sugars from LTA.

**Fig 4:**
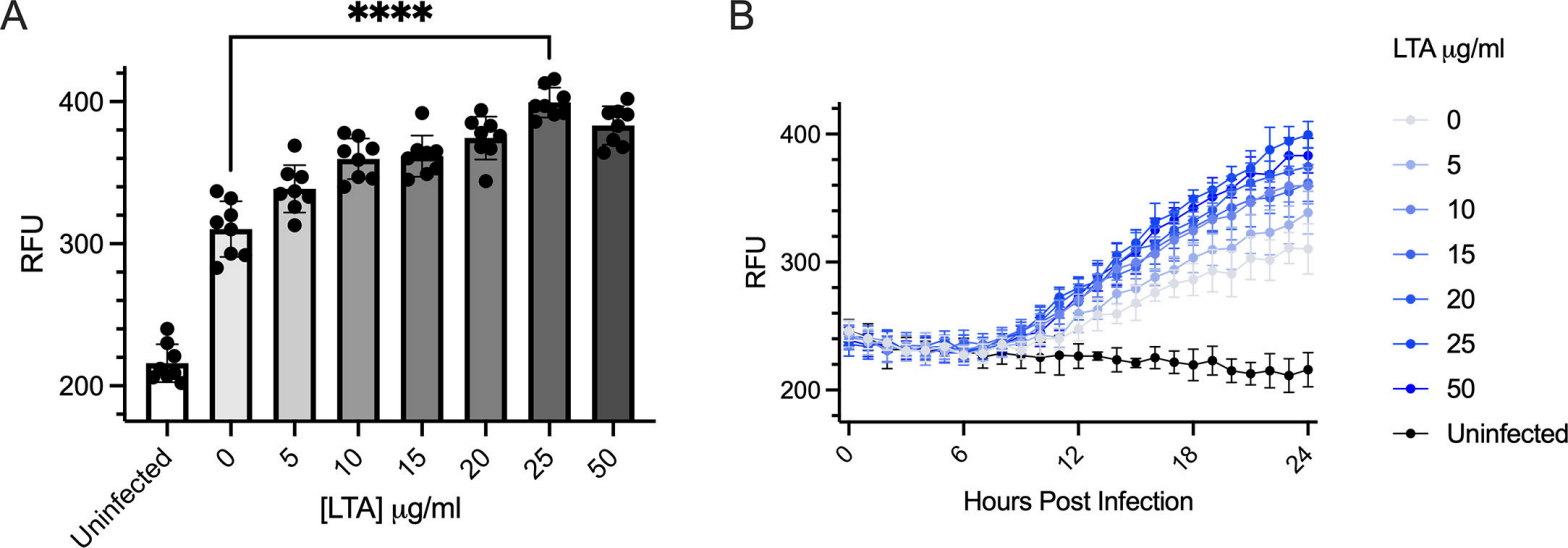
LTA increases viral infection of host cells. (**A**) Relative fluorescence units (RFU) of cells infected with mNeon-PR8 in the presence of the indicated concentration of LTA after 24 h. ****P < 0.0001 by unpaired *t*-test. (**B**) Time course of viral infection with mNeon-PR8 from 0 to 24 h in the presence of indicated concentration of LTA.

Given that LTA likely interacts with influenza surface proteins involved in infection, we asked whether LTA could impact viral infectivity. We devised a fluorescence-based assay using a reporter influenza strain, PR8-mNeon (36). Infected cells express the mNeon protein, allowing for live tracking of infectivity by plate reader. PR8-mNeon was pre-incubated with LTA from *S. pyogenes*, at concentrations ranging from 0 to 50μg/mL, before infection of MDCK cells in a 96-well format at various concentrations. Unlike what has been demonstrated for LPS, causing a decrease in IAV infectivity (27), LTA appears to increase infectivity. In a dose-dependent manner, reporter virus fluorescence increased with LTA concentration, with 25 µg/mL of LTA achieving the most substantial effect at the 24 hour timepoint (Fig. 4A). When examining fluorescence over time, incubation with LTA does not appear to affect the timing of the infection, instead just increasing the amount of viral protein expression (Fig. 4B).

## DISCUSSION

Here, we suggest that teichoic acid is serving as one of the ligands for adherence of IAV particles to pneumococcal cells via competitive inhibition of viral binding and genetics-based approaches. As *tacL* deletion strains have previously been shown to demonstrate altered lipoprotein expression and membrane fluidity (37), we cannot rule out the impact of those additional membrane changes also playing a role in altered viral binding. Further, it was recently shown that a Δ*tacL* mutant pneumococcus had increased surface expression of teichoic acids, attached to lipid II (38). Therefore, it is possible that the decreased binding we demonstrate in the Δ*tacL* mutant is due to abnormally high levels of teichoic acid masking the true binding partner for IAV. However, we do demonstrate *in vitro* interactions of the viral neuraminidase (NA) protein and possibly hemagglutinin (HA) protein with purified LTA, which suggests that a direct interaction does occur.

We demonstrate interaction between viral particles and LTA and/or bacterial cells by three complementary methods: measuring viral binding directly through amount of viral genome associated with bacterial cells, amount of viral protein produced as measured in a reporter assay, and viral infectivity as measured by successful production of progeny viruses. The combination of these three methods increases the robustness of our results.

These data fit into the growing body of knowledge around bacterial glycans mediating trans-kingdom interactions during infection. Enteric viruses have been demonstrated to have enhanced infectivity of host cells in the presence of bacterial glycans (22–24, 26). Likewise, we demonstrate a trend toward increased viral infectivity in the presence of LTA (Fig. 4). There are a few potential causes for this increase in infectivity. LTA-coated viral particles may adhere more readily to MDCK cells, as per our hypothesis. Alternatively, the LTA may induce an innate cell response, facilitating viral infection. Other studies (27, 39) with IAV and bacterial glycans have identified interactions between the Gram-negative outer membrane glycan lipopolysaccharide (LPS) and IAV. However, these interactions were detrimental to IAV and reduced infectivity. The detrimental interactions for IAV occurred with LPS from microbes commonly isolated from the mammalian gastrointestinal tract, while the neutral or perhaps positive interactions reported here were with LTA from microbes commonly isolated from the upper respiratory tract, the natural infection site for human IAV. This supports a role of the microbiota in tissue tropism of IAV.

This study uses a single subtype of IAV: H1N1. While direct interactions were seen between an H3N2 strain and *S. pneumoniae* (13), it is possible that interactions between H3N2 subtype virus and *S. pneumoniae* are mediated by other bacterial factors, and that the findings from this study are not generalizable to all pneumococcal-IAV interactions.

This study is additionally limited by the use of *in vitro*, non-host relevant conditions. During natural infection, or immediately following excretion by the host during a transmission event, it is possible that the native sialylated host glycoproteins outcompete the bacterial glycans for IAV binding, and these bacterial-viral interactions would not be demonstrated. Further, the use of *in vitro* conditions means that the bacterial cell envelope is not being modified in response to sensing of host signals. During infection, the amount of capsule varies (40), and therefore, the amount of LTA visible to the virus will also vary. *In vivo* modifications of teichoic acid directly include D-alanylation of the teichoic acid (41) and altered levels of phosphocholine on the teichoic acids (42). The PG can be directly modified through the addition (43) and removal (44) of acetyl groups on the disaccharide subunit of PG. These modifications could either enhance or reduce the levels of IAV binding compared to what we demonstrate *in vitro*.

Overall, this study adds to the growing field of knowledge on bacterial-viral interactions by identifying one mechanism for binding of IAV particles to pneumococcal cells. Insights gained from this work increase our understanding of the co-pathogenesis of *S. pneumoniae* and influenza, as well as the enhanced pathogenesis of the co-infection.

## MATERIAL AND METHODS

### Bacterial culture

*S. pneumoniae* strains BHN97 or TIGR4 were grown on Tryptic Soy Agar (BD 212305) supplemented with 3% sheep red blood cells (Lampire) in a humidified incubator at 37°C with 5% CO_2_ atmosphere. Overnight cultures were transferred to Todd Hewitt media (BD 249240) supplemented with 0.2% yeast extract (VWR J850) (THY media) or to C+Y (45) media and grown to mid-log growth phase (OD_620_ ~0.400) in a humidified incubator at 37°C with 5% CO_2_ atmosphere.

### Viral culture

Influenza virus strain A/Puerto Rico/08/1934 (H1N1) and PR8-mNeon (H1N1) (36) were grown in Madin-Darby Canine Kidney cells (ATCC CCL-34) in Minimal Essential Media (Gibco) supplemented with GlutaMax (Gibco), sodium pyruvate (Gibco), and 10% fetal bovine serum (Hyclone) to >90% confluency. Prior to infection, cells were washed twice with PBS. Cells were infected at an MOI of ~0.01 in Minimal Essential Media (Gibco) supplemented with BSA fraction V (Gibco), GlutaMax (Gibco), and 1 ug/mL TPCK-Trypsin (Thermo Scientific 20233). After 96 h of infection and complete destruction of the monolayer, supernatant was harvested, centrifuged at 5000 × *g* to pellet debris, and was aliquoted and stored at −70°C. Titer was determined by 50% tissue culture infectious dose (TCID_50_) (see method below).

Influenza strain A/California/04/2009 (H1N1) was cultured as follows. MDCK cells were plated to confluency in a 6-well format in Eagle’s minimum essential medium with L-glutamine (Quality Biological) supplemented with 10% FBS (Corning). Wells were washed with PBS and infected at a ~ 0.1 multiplicity of infection (MOI) in 400 uL of EMEM with L-glutamine supplemented with 0.075% BSA (Sigma). After 1 h of infection, 2 mL of additional EMEM with BSA supplemented with 1 µg/mL TPCK Trypsin (Worthington Biochemical) was added. After 24 h, supernatant was spun down at 10,000 × *g* and frozen at -80°C. Viral stock titers were determined by TCID_50_.

### Generation of bacterial mutants

#### tacL (SP_1893) and lytR (SP_1942) deletions

Gene deletion was performed by allelic exchange with an erythromycin cassette. Approximately 1 kb upstream and downstream of the gene of interest was amplified with primers (see Table 1) incorporating an overlap of the erythromycin resistance cassette using PrimeStar High-Fidelity Polymerase (Takara). PCR products were gel extracted (Qiagen MinElute) and, along with the erythromycin resistance cassette, were combined using splicing by overlap extension PCR using PrimeStar High-Fidelity Polymerase (Takara). Purified PCR product was transformed into BHN97 or TIGR4, in THY media supplemented with 0.002% BSA, 0.2% glucose, and 0.0002% CaCl_2_ (46). Cultures were grown to early log (OD_620_ approximately 0.2), diluted 1:100 in supplemented THY, and incubated for 14 min with 2 µg/mL CSP-1 and CSP-2 for BHN97, or CSP-2 for TIGR4 or T4R (47). Gene deletion was confirmed by the absence of amplification with internal primers.

**TABLE 1:** Primers used in this study

Primer name	Sequence	Citation
tacL up F	GTAGTGGGTAAATCCACTATAG	This study
tacL up R	GAGTCGCTTTTGTAAATTTGGGTTTTATAAGTTTGAAATCTTCGGC	This study
Erm F	GGAAATAAGACTTAGAAGCAAAC	
ErmR	CCAAATTTACAAAAGCGACTC	
tacL down F	GTTTGCTTCTAAGTCTTATTTCCAATGAATCCTTTCTCTCCAAATCAG	This study
tacL down R	GAGATGCTATTGGAGGAGTTC	This study
tacL internal F	GCCAGTTTAGAACATTTCCAAATTGTG	This study
tacL internal R	CCGTCATGTCCGATACCAAC	This study
lytR up F	CCAATGACCATTGTCTCACTTC	This study
lytR up R	GTTTGCTTCTAAGTCTTATTTCCATTTCTACTAACCTATCAGTTTACC	This study
lytR down F	GAGTCGCTTTTGTAAATTTGGTTAACTTTTGATACAAATAAAAAAATCAATCG	This study
lytR down R	CCTACATTCTAATGATCCTCAG	This study
lytR internal F	GGAATGGTGCTAGCTTTACTTTC	This study
lytR internal R	CCAACATATGTGCTCTCGAAAC	This study
CEP insert up FWD	GCAGAATCTCCCAAGGAAAG	(48)
CEP insert up REV-SPEC	GAAAACAATAAACCCTTGCATATGGTGATAGGATAGGAAGCATC	(48)
CEP insert amiF FWD	CAC CTT TTT CAT CAC CTG TC	(48)
CEP insert amiF REV	CG TTT CCC TTG AAC TAG TCG AAG	(48)
Spec-TacL comp FWD	GTATTCACGAACGAAAATCGATCGGGTAATTGAGAATGGAG	This study
TacL comp-amiF REV	GAC AGG TGA TGA AAA AGG TGT TAA TCC GTC ATG TCC GAT AC	This study
Spec F	atcgattttcgttcgtgaatac	
Spec R	CATATGCAAGGGTTTATTGTTTTC	
Inf A forward	GACCRATCCTGTCACCTCTGAC	(49)
Inf A reverse	AGGGCATTYTGGACAAAKCGTCTA	(49)
Inf A probe (FAM, BHQ1)	TGCAGTCCTCGCTCACTGGGCACG	(49)
lafB Up F	gaccaagctcgtaccatccg	This study
lafB Up R	GAATTTGTAAAGTTAATTGcctgtcctgctactttc	This study
PhunSweet	CAATTAACTTTACAAATTCCCACTATTAAGG	(50)
ERM_R	CCAAATTTACAAAAGCGACTC	(50)
lafB Down F	GCTTTTGTAAATTTGGgaagagcatctgttacaaatctggttgg	This study
lafB Down R	ccaaacattttatcactgatgaggttgttc	This study
lafB F	caagtgagaaagtagcaggacag	This study
lafB R	ctccctaaagcggcttgtttc	This study

#### tacL complement

The *tacL* gene and upstream region containing the probable promoter were inserted in an ectopic locus, the CEP locus (30), as described previously (48) and transformed into *S. pneumoniae* as described above. Due to the lack of recovery of transformants when the *tacL* deletion strain was transformed with the construct containing the *tacL*-complement PCR product, wild-type BHN97 and TIGR4 were transformed with the *tacL*-complement PCR product. Once insertion was confirmed, those strains were transformed with genomic DNA from the respective *tacL* deletion strain to generate the *tacL*-complement.

#### lafB deletion

The lafB mutant was generated in strain TIGR4 by insertional mutagenesis with the PhunSweetErm cassette (50). One kilobase fragments upstream and downstream of the *lafB* gene were amplified using the primers indicated in Table 1 and Takara Ex Taq. The first and last ~30–60 bp of *lafB* were left intact to avoid disrupting neighboring genes. The purified fragments (extracted with Qiagen MinElute) were combined with the PhunSweetErm cassette via overlap extension PCR, again using Takara Ex Taq. The final product was transformed into TIGR4 grown in C + Y to an OD620 of 0.07. The transformation proceeded for a few hours in fresh C + Y supplemented with 3 µg/mL CSP-2 before selecting on erythromycin. The mutant was validated by PCR.

### Competitive binding with bacterial glycans

*Bacillus subtilis* peptidoglycan (Sigma 69554) and *Streptococcus pyogenes* lipoteichoic acid (Sigma L3140) were resuspended in sterile deionized water at a concentration of 1 mg/mL. Twenty microliters of the glycan, or equivalent volume of water, were mixed with 20 µL of a 7 log TCID_50_/mL stock of IAV strain PR8 brought to a total volume of 100 µL in PBS. Samples were incubated with rocking at 37 C for 30 min. Ten microliters of the sample were added to 10^7^ CFU washed mid-log *S. pneumoniae* in a volume of 1 mL. Residual virus-alone samples were frozen at −70°C to determine if incubation with bacterial glycan reduced infectivity of IAV. Samples were incubated with rocking at 37°C for 30 min. Samples were centrifuged at 10,000 × *g* for 3 min, washed once in PBS, and resuspended in 100 µL of 10× suggested working concentration of PenStrep (1,000 U/mL penicillin, 1,000 U/mL streptomycin) (Gibco). Samples were frozen at −70°C until titer was determined by TCID_50_. Each strain had at least six biological replicates performed in at least two independent experiments.

### Viral binding by qPCR

*S. pneumoniae* strain TIGR4 was grown to mid-log phase in C+Y (~0.4 OD620). One milliliter aliquots of culture were spun down at 6,000 × *g* for 5 min. Bacterial pellets were resuspended in 1 mL PBS containing 10^6.5 TCID50s of influenza stock (A/California/04/2009) and lipoteichoic acid sourced from *Streptococcus pyogenes* (Sigma Aldrich L3140) in a range of concentrations from 0 to 10 μg/mL. For tube-binding and influenza-positive controls, the same volume of virus was added to a tube without bacteria. Bacterial/viral mixtures were incubated at 37°C for 30 min, rotating end-over-end. Samples were spun down, washed with 1 mL PBS, spun down again, and frozen at -80°C.

Influenza RNA was extracted from the bacterial pellets using an altered protocol for the RNeasy Kit (Qiagen), including the provided RLT, RW1, and RPE buffers. Bacterial pellets were resuspended in 50 µL of PBS and were transferred to another tube containing 350 µL RLT buffer supplemented with 1% 2-mercaptoethanol (Sigma) by volume. Virus-only samples were similarly resuspended in 50 µL PBS, except that a bacterial pellet of TIGR4 was added. The bacteria ensured proper extraction and normalization of viral RNA. Viral samples were combined with RLT containing 2-mercaptoethanol as well. Samples were lysed using lysing matrix E silica beads (MP Biomedicals), pulsing with the Fast Prep (MP Biomedicals) for three 45-second cycles. Samples were heated to 70°C for 10 min. Two hundred microliters of lysate were transferred to a Qia-shredder (Qiagen) column before spinning at maximum speed for 1 min. The flow-through was combined with an equal volume of 70% ethanol before applying to an RNeasy mini column. Columns were spun at 10,000 × *g* for 30 s, discarding the flow-through. Three hundred fifty microliters of RW1 buffer were run through the columns twice, followed by two rounds of 500 uL of RPE buffer. Columns were dried by spinning for 2 min at 10,000 × *g*. RNA was eluted in two batches of 35 µL RNase-free water (Invitrogen).

TaqMan Fast Virus 1-Step Master Mix (Applied Biosystems) was used with the listed primers (Table 1) to quantify the influenza RNA content by qPCR. The average Ct for the 0 µg/ml LTA samples was set to a value of 1. All other samples were assigned relative to this average, with two corresponding to twice as much viral RNA, 0.5 for half, and so on.

### Fluorescent infectivity assay

5.5 TCID50 of MDCK-grown mNeon PR8 (36) was incubated with lipoteichoic acid (LTA) from *Streptococcus pyogenes* at a range of concentrations from 5 to 50 µg/mL in a total of 1 mL infection media (EMEM with L-glutamine [Quality Biological] +0.075% BSA [Sigma]). Virus/LTA mixtures were incubated at 37°C rotating end-over-end for 30 min. A black, clear-bottom, tissue culture-treated 96-well plate of confluent MDCK cells was washed with 200 µL PBS per well. Wells were infected with 100 µL of virus/LTA mixture, with eight replicates per LTA concentration. The infection proceeded at 37°C + 5% CO2 in a Cytation3 plate reader, taking fluorescence endpoints every hour for 24 h. Excitation was set to 485 nm, emission to 528 nm, and the gain to 75.

### IAV binding to bacterial mutants

Five log TCID_50_ IAV strain PR8, in 10 µL volume, was added to 10^7^ CFU washed mid-log bacteria resuspended in 1 mL PBS and was incubated at 37°C with rocking for 30 min. Samples were centrifuged at 10,000 × *g* for 3 min, washed once in PBS, and resuspended in 100 µL of 10× suggested working concentration of PenStrep (Gibco) (1,000 U/mL penicillin, 1,000 U/mL streptomycin). Samples were frozen at −70°C until titer determined by TCID_50_. Each strain had at least six biological replicates performed in at least two independent experiments.

### Determination of TCID_50_

Viral titer was determined by 50% tissue culture infectious dose assay. MDCK cells were seeded at 3 × 10^4^ cells per well in 96-well plates (Costar). Serial 10-fold dilutions of rehydrated samples were made in infection media. Cells were infected in triplicate with each dilution. Seventy-two hours post-infection, supernatant from infected cells was mixed 1:1 with 0.5% turkey red blood cells in PBS (Lampire) in V-bottom 96-well plates for hemagglutination assay. Plates were incubated at room temperature for 30 min. Hemagglutination was read for each well, and 50% tissue culture infectious dose was calculated using the method of Reed and Munch (51).

### LTA-pulldown

EZ-Link NHS Biotin (Thermo) was dissolved in DMSO to a concentration of 3.4 mg/mL. Fifty microliters of biotin were incubated with 50 uL of 5 mg/mL LTA from *S. pyogenes* (Sigma Aldrich L3140) on ice for a few hours to allow biotinylation. The biotinylated LTA was desalted into PBS using a NAP-5 column (Cytiva), yielding an end product of 0.5 mg/mL.

Samples were prepared by combining 1 µg His-tagged hemagglutinin from A/California/04/2009 (Sino Biologicals) or His-tagged neuraminidase from A/California/04/2009 (Sino Biologicals) with 5 µg biotinylated LTA in PBS, for a total volume of 500 µL. An irrelevant protein control was prepared with 1 µg His-tagged human GAPDH (Sino Biological). Additionally, negative controls lacking LTA were prepared. Samples were incubated rocking for three and a half hours at 4°C.

Dynabeads MyOne Streptavidin T1 beads (Invitrogen) were prepared by washing 25 µL aliquots of beads with 1 mL PBS three times. Beads were blocked with 1 mL 5% BSA in PBS, rocking for at least 30 min at 4°C. Block was removed from the beads, and beads were incubated with the protein/LTA mixtures for 30 min, rocking at 4°C. Beads were washed five times with 1 mL PBS prior to freezing at -80°C.

Samples were eluted from the beads by heating to 99°C in SDS-PAGE loading buffer containing 2-mercaptoethanol. Samples were analyzed using traditional Western blot methods. The primary antibodies were MA5-36006 for anti-HA (Invitrogen), GTX125974 for anti-NA (GeneTex), and MA5-15738 for anti-GAPDH (Invitrogen). The secondary was goat anti-mouse IgG HRP Conjugate, Cat #1706516 (Bio-Rad Laboratories), or goat anti-rabbit IgG HRP Conjugate, Cat #1706515 (Bio-Rad Laboratories). Blots were processed with SuperSignal West Dura Extended Duration Substrate (Thermo Scientific, Ref. 34076).

### Statistical analyses

Statistical analyses were performed using GraphPad Prism Version 10.4.1. Data were tested for normality using the Kolmogorov-Smirnov test. If data were normal, unpaired *t*-tests were performed; if data were non-normal, the Mann-Whitney test was performed for pairwise comparisons. Correlation of relative viral binding with LTA concentration was determined by Spearman’s r and was found to be significant.

## SUPPLEMENTAL MATERIAL

Figure S1 msphere.00504-25-s0001.docx LTA/influenza binding competition by cosedimentation.

Figure S2 msphere.00504-25-s0002.docx Uncropped blots.

ASM does not own the copyrights to Supplemental Material that may be linked to, or accessed through, an article. The authors have granted ASM a non-exclusive, world-wide license to publish the Supplemental Material files. Please contact the corresponding author directly for reuse.
