# Supplementary material for: Lipoteichoic acid mediates binding of Streptococcus pneumoniae and influenza A virus
Source: mSphere. 2025 Nov 28;10(12):e00504-25. doi: 10.1128/msphere.00504-25 (PMC12724309; doi:10.1128/msphere.00504-25)
Supplement: Figure S1 — LTA/influenza binding competition by cosedimentation. [file msphere.00504-25-s0001.docx]

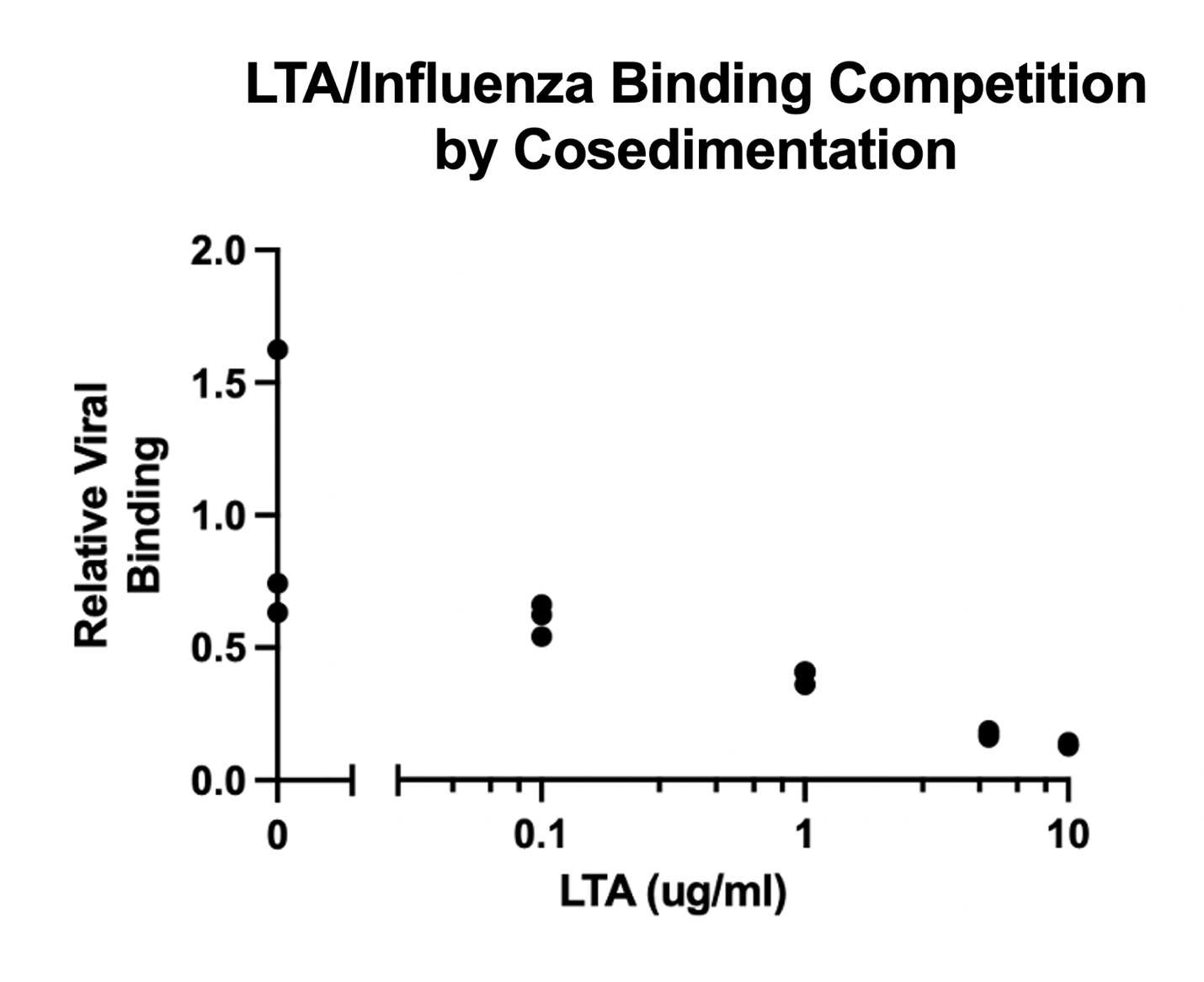


**Supplemental Figure 1: LTA/Influenza Binding Competition by Cosedimentation.** IAV strain A/California.04/2009 was pre-incubated with *S. pneumoniae* strain TIGR4 in the presence of indicated concentration of *S. pyogenes* LTA and bound IAV assessed by qRT-PCR for viral genomic RNA relative to the 0 LTA control. Each point represents a single sample.
