# Supplementary material for: Lipoteichoic acid mediates binding of Streptococcus pneumoniae and influenza A virus
Source: mSphere. 2025 Nov 28;10(12):e00504-25. doi: 10.1128/msphere.00504-25 (PMC12724309; doi:10.1128/msphere.00504-25)
Supplement: Figure S2 — Uncropped blots. [file msphere.00504-25-s0002.docx]

(A)

(B)

(C)

**Supplemental Figure 2: Uncropped blots.** Biotinylated *S. pyogenes* LTA was incubated with: (A) hemagglutinin (HA) from A/California/04/2009 (H1), (B) neuraminidase (NA) from A/California/04/2009 (N1), or (C) a non-specific protein: GAPDH, followed by pulldown with streptavidin beads, and detected by Western Blot. (L) = SeeBlue™ Plus2 Pre-stained Protein Standard (Invitrogen™). (+) = purified protein alone. (Biotin-LTA) = pulldown of purified protein after interaction with biotinylated LTA. (Beads alone) = pulldown of purified protein with beads alone.
